# Royal College of Physicians of Edinburgh

**Published:** 1891-03

**Authors:** G. A. Gibson

**Affiliations:** Secretary. March 9th, 1891


### Royal College of Physicians of Edinburgh.

### PARKIN BEQUEST.

In terms of the Bequest made to the Royal College of Physicians of Edinburgh by the late Dr. John Parkin, Fellow of the College, a prize is hereby offered for the best Essay—

> "On the Curative Effects of Carbonic Acid Gas or other forms of Carbon in Cholera, the different forms of Fever, and other Diseases."

The prize is of the value of One Hundred Pounds Sterling, and is open to competitors of all nations.

Essays intended for competition, which must be written in the English language, to be received by the Secretary not later than 31st December, 1892. Each Essay must bear a motto, and be accompanied by a sealed envelope bearing the same motto outside and the author's name inside.

The successful candidate must publish his Essay at his own expense, and present a printed copy of it to the College within the space of three months after the adjudication of the prize.

In name and by authority of the Council of the College,

G. A. GIBSON, M.D.,

*Secretary.*

*March 9th*, 1891.

